# Evaluation of MAGLUMI syphilis test for accurate detection of syphilis antibodies in blood donors and suspected syphilis cases

**DOI:** 10.3389/fcimb.2025.1578060

**Published:** 2025-05-15

**Authors:** Wei Fang, Yun Zhang, Min Shi, Yuqin Liao, Lanhuan Peng, Hailin Zhong, Jun Yin, Taoran Mo, Heng Li, Zhonggang Fang

**Affiliations:** ^1^ Laboratory Medicine, Guangdong Provincial People’s Hospital (Guangdong Academy of Medical Sciences), Southern Medical University, Guangzhou, China; ^2^ Research & Development Department, Shenzhen New Industries Biomedical Engineering Co., Ltd. (Snibe), Shenzhen, China; ^3^ Faculty of Biology, Shenzhen MSU-BIT University, Shenzhen, China

**Keywords:** syphilis, MAGLUMI, chemiluminescence immunoassay, diagnosis, Treponema pallidum

## Abstract

**Background:**

The diagnosis of syphilis is critical to initiate treatment at an early stage and control the syphilis epidemic. The serological detection of treponemal antibodies is recommended in the reverse sequence screening algorithm as the first screening test.

**Study design:**

Serum samples from 5,081 unselected blood donors and 213 hospitalized patients were collected to evaluate the diagnostic specificity. To assess the diagnostic sensitivity, 487 positive samples were collected. 405 cross-interference samples were tested to evaluate analytical specificity. All samples were tested with the MAGLUMI Syphilis (Chemiluminescence immunoassay, CLIA) Test and the obtained results were compared with the Abbott ARCHITECT Syphilis TP reference test.

**Results:**

The diagnostic specificity and sensitivity of the MAGLUMI Syphilis (CLIA) Test was 99.96% (95% CI, 99.87-99.99%) and 100.00% (95% CI, 99.22-100.00%), respectively. The analytical specificity and the analytical sensitivity of the Test was 100.00% and 2.529 mIU/ml, respectively. No significant interference and cross-reactivity were observed in a number of potential factors.

**Conclusions:**

The performance of the MAGLUMI Syphilis (CLIA) Test makes it suited for identification of treponemal antibodies in screening populations as well as patients presenting with suspicion of syphilitic infection.

## 1 Introduction

Syphilis is a sexually and vertically transmitted bacterial infection caused by the spirochete *Treponema pallidum* (*T. pallidum*) subspecies *pallidum* (Peeling et al., 2017; Peeling et al., 2023). If left untreated, the disease lasts for many years and is divided into several stages. Early syphilis includes primary syphilis, secondary syphilis and early latent syphilis. And late syphilis includes late latent syphilis and tertiary syphilis (neurosyphilis and cardiovascular syphilis) (Kingston et al., 2016). The incidence of syphilis has increased over the past few years, especially among men who have sex with men (MSM), probably due to the reestablishment of sexual networks and increased frequency of sexual contact (Kenyon et al., 2014). Between 2016 and 2023, the global number of reported cases of congenital syphilis has been increasing rapidly (Sankaran et al., 2023). In 2022, an estimated 8 million adults aged 15 to 49 acquired syphilis and approximately 700,000 congenital syphilis globally (WHO, 2023). In 2022, the World Health Organization (WHO) released its new strategy for the prevention and treatment of sexually transmitted infections 2022-2030. The strategy focuses on eliminating congenital syphilis in selected populations such as pregnant women by comprehensive syphilis screening and treatment, with the dual goal of reducing global syphilis incidence by 90 percent and congenital syphilis cases to 50 or fewer per 100,000 live births by 2030 (WHO, 2022).

Although syphilis can be cured with penicillin, the challenge is that many infections are unrecognized due to the difficulty of clinical diagnosis of syphilis (Peeling et al., 2017). If left untreated, around 25% of patients will develop tertiary syphilis, which can lead to severe complications such as brain and cardiovascular diseases (Jankowska et al., 2022). Therefore, it is crucial to detect syphilis infection early and provide patients with timely treatment to prevent these complications and improve patient health. A syphilis vaccine may be a complementary approach to prevent infection especially in countries with limitation of diagnosis and treatment. Although several vaccine prototypes were proved to slow the disease progression, no effective vaccine has yet been developed (Avila-Nieto et al., 2023). The current testing algotithms used for syphilis diagnostics typically rely on a combination of nontreponemal and treponemal serologic tests (Papp et al., 2024). Nontreponemal tests are an indirect method using lipoidal antigens to detect a mixture of heterophile IgG and IgM formed by a concomitant *T. pallidum* infection or another condition related to host tissue damage and release of lipoidal antigens. Treponemal tests detect an antibody response to antigens specific to *T. pallidum*. The traditional algorithm for syphilis serologic screening begins with a nontreponemal test, and any reactive specimens are tested for confirmation by a treponemal test. This algotithm has been widely used for decades due to the rapid, cheap and simple characteristics of nontreponemal test (Papp et al., 2024). However, the establishment of automated treponemal immunoassays is leading to the reverse algorithm for syphilis screening, which might be more sensitive in detecting early or late latent syphilis, but an increase in false positives might occur in low-prevalence populations (Papp et al., 2024). The procedure of the reverse screening strategy recommended by the European Center for Disease Control and Prevention (ECDC) is: first use a *T. pallidum* antibody test, continue to use another different *T. pallidum* antibody test as a confirmatory test for positive samples, and then use non-*T. pallidum* antibody test to evaluate syphilis activity and treatment effect (Janier et al., 2021).

If untreated, syphilis in pregnancy can lead to serious adverse outcomes including congenital syphilis, preterm birth, stillbirth, and neonatal death (Qin et al., 2014). Congenital syphilis is also the second major cause of stillbirths that can be prevented worldwide after malaria (WHO, 2021). Even though the incidence of adverse pregnancy outcomes has decreased significantly after treatment in pregnancies with syphilis, the risk remains higher than uninfected pregnancies (Rac et al., 2017). Therefore, universal syphilis testing for high-risk women such as pregnancies was recommended by most guidelines (Trinh et al., 2019). Treponemal tests occasionally produce false-positive results in autoimmune diseases, Lyme disease and pregnant women (Janier et al., 2021). A study involving 94,462 patients found a false positive rate of 0.62% (588 patients), with the majority of these cases observed in older adults over 60 years of age who had a history of malignancy (Ishihara et al., 2021). Therefore, it is of great clinical significance to verify the analytical specificity of treponemal tests in these specific populations.

The outer membrane proteins of *T. pallidum* are scarce, but there are abundant lipoproteins between the inner membrane and peptidoglycan, mainly TpN47/TpN15/TpN17/TmpA (TpN44.5). Its recombinant antigen is the main diagnostic antigen of syphilis serology at present (Silva et al., 2020). The MAGLUMI Syphilis test (Snibe, Shenzhen, China) is a chemiluminescent immunoassay (CLIA) that has recently been developed to detect antibodies to *T. pallidum*. The assay is based on the double-antigen sandwich principle, using a recombinant core antigen combined with the TpN15, TpN17, and TpN47 antigens. This antigen combination is widely applied in approval tests including Abbott ARCHITECT Syphilis TP and Roche Elecsys Syphilis (Park et al., 2020). In this study we evaluated the clinical performance of the test to verify whether the test is suited for syphilis.

## 2 Material and methods

### 2.1 MAGLUMI syphilis (CLIA) and reference tests

The MAGLUMI Syphilis (CLIA) Test is a sandwich chemiluminescence immunoassay that uses a recombinant *T. pallidum* antigen labeled by fluorescein isothiocyanate (FITC) and a biotinized recombinant *T. pallidum* antigen to react with a sample to form a sandwich complex. Subsequently, N-(4-aminobutyl) -n-ethylisolumitol (ABEI) labeled sheep anti-FITC polyclonal antibody and magnetic microbeads coated with streptavidin were added to bind the solid through the interaction of biotin and streptavidin. After precipitation in a magnetic field, Starter 1 + 2 is added to initiate a chemiluminescence reaction and the light signal is measured through a photomultiplier tube. All samples in this study were tested according to the manufacturer’s instructions using the MAGLUMI Syphilis (CLIA) test on Snibe MAGLUMI Platform including X8 and 4000 Plus. The results were validated and compared with reference tests. Blood donor samples were compared with Abbott ARCHITECT Syphilis TP, and inconsistent samples were confirmed with Roche Elecsys Syphilis. Syphilis positive samples were tested using Abbott ARCHITECT Syphilis TP and Roche Elecsys Syphilis.

### 2.2 Blood donor and patient samples

**Figure 1:**
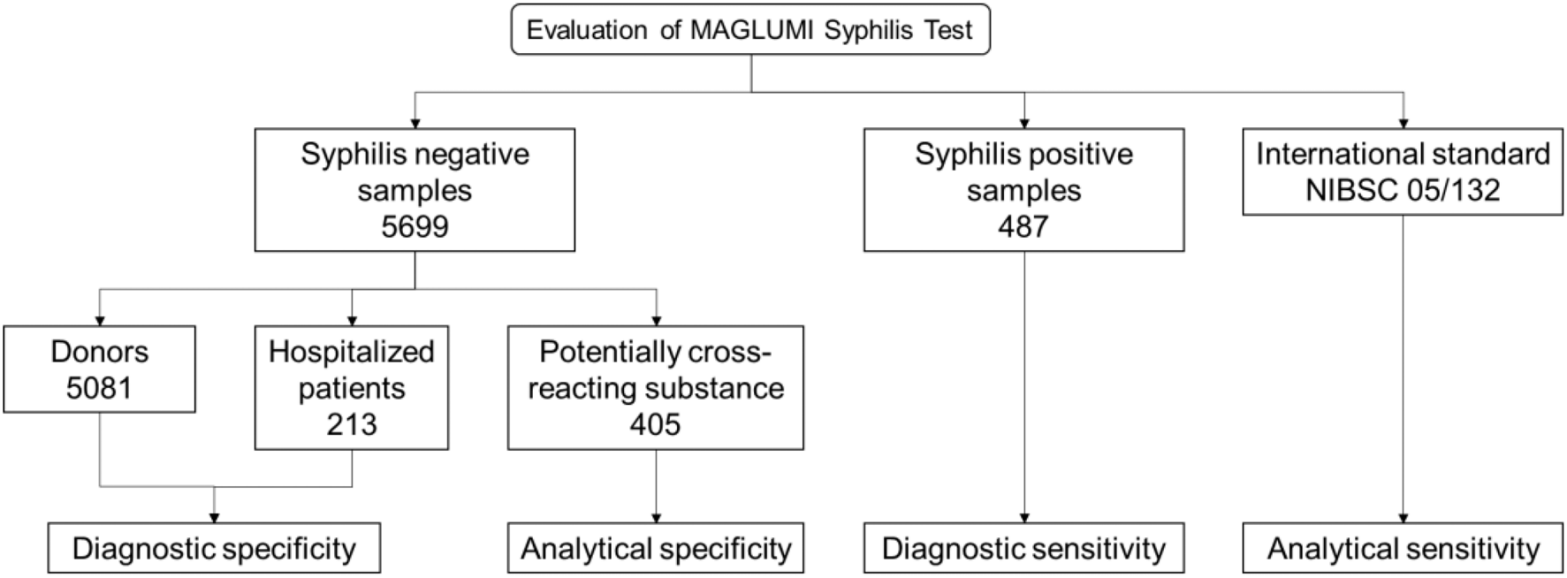
Study flow diagram.

A total of 5,081 donor samples (serum or plasma) were collected from two blood donation centers in Germany, where one of the reference tests found two negative or false response to syphilis antibodies (**Figure 1**). All samples have been tested for MAGLUMI Syphilis (CLIA). Samples with results greater than or equal to 1 AU/mL (≥1 AU/mL) were considered suspicious and the test was repeated twice. After retesting, samples with at least 1 repeat reaction result were tested for syphilis confirmation.

A total of 487 syphilis positive samples were selected based on prior test results generated by both Abbott ARCHITECT Syphilis TP and Roche Elecsys Syphilis (235 samples were collected from Germany and 252 samples were collected from China), of which 103 samples with known stages of syphilis (**Figure 1**). Among the samples with known stages, there were 49 cases of primary syphilis, 19 cases of secondary syphilis, and 35 cases of tertiary syphilis.

Syphilis negative samples from 213 hospitalized patients who had a medical condition and were taking medication were evaluated (**Figure 1**).

### 2.3 Cross reactivity and endogenous interference

In total 405 samples with potentially cross-reacting substance (111 samples were collected from Germany and 294 samples were collected from China) and general endogenous interfering substances including hemolytic (1.0 g/mL), lipemic (2.0 g/dL) and bilirubin (40.0 mg/dL) were evaluated (**Figure 1**).

### 2.4 International standard

The analytical sensitivity of MAGLUMI Syphilis (CLIA) was evaluated using the international standard NIBSC 05/132 (**Figure 1**).

### 2.5 Statistical analysis

Excel 2019 (Microsoft Inc., USA) was used to calculate average value, standard deviation, coefficient of variation, proportions and corresponding Wilson 95% confidence intervals. GraphPad Prism 9.0 (GraphPad Software, La Jolla, CA) was used.

### 2.6 Ethics statement

The clinical performance evaluation study was conducted by a third-party organization (laboratories of Biomex GmbH, Heidelberg, Germany) and Guangdong Provincial People’s Hospital (Guangzhou, P. R. China) in accordance with the guidelines of the Declaration of Helsinki. The samples used in this study were all residual samples with extensive informed consent and ethical approval (Ethics No.: KY2024-533-01). All residual serum samples were collected and tested from these two study centers (in Germany from July 2023 to February 2024, in China from July 2024 to August 2024).

## 3 Results

### 3.1 Diagnostic specificity and sensitivity

2 of the 5,699 syphilis negative samples gave reactivity results for the MAGLUMI Syphilis (CLIA) test (Information on the two discordant samples is provided in the **Supplementary Table 1**). Samples of 213 hospitalized patients did not respond to the MAGLUMI Syphilis (CLIA) test. These resulted in a diagnostic specificity of 99.96% (5697/5699) (**Table 1**). All 487 samples that were antibody positive for *T. pallidum*, including 103 with known stages of syphilis, were tested positive for MAGLUMI Syphilis (CLIA) with a diagnostic sensitivity of 100.00% (**Table 2**).

**Table 1:** Performance of syphilis screening of the MAGLUMI syphilis (CLIA) test on blood donor samples and hospitalized patients.

Measure	Calculation	Estimate	95%CI
Specificity	5697/5699	99.96%	99.87~99.99%
Sensitivity	487/487	100.00%	99.22~100.00%
FPR	2/5699	0.04%	0.01~0.14%
FNR	0/487	0.00%	0.00~1.73%
PPV	487/489	99.59%	98.52~99.89%
NPV	5697/5697	100.00%	99.93~100.00%
Accuracy	6184/6186	99.96%	99.86%~99.99%

FPR, false positive rate; FNR, false negative rate; PPV, positive predictive value; NPV, negative predictive value; CI, confidence interval.

**Table 2:** Sensitivity of the MAGLUMI^®^ Syphilis (CLIA) Test in Syphilis positive samples.

Diagnostic sensitivity evaluation	Stages	samples	reactive	Non-reactive
Syphilis positive samples	Unknown stages	384	384	0
	Primary	49	49	0
	Secondary	19	19	0
	Tertiary	35	35	0
Total		487	487	0
Sensitivity	100%			
95% CI	99.22~100.00%			

### 3.2 Analytical specificity

None of the 405 potentially cross-reacting samples reacted with the MAGLUMI Syphilis (CLIA) test (**Table 3**). The analytical specificity of 405 samples with potential cross-reactive substances was 100.00% (405/405). No interference occurs at given concentrations of general endogenous interfering substances including hemolytic (1.0 g/mL), lipemic (2.0 g/dL) and bilirubin (40.0 mg/dL).

**Table 3:** Analytical specificity of MAGLUMI syphilis (CLIA) test in samples with potentially cross-reacting substances.

Cross-reacting substances	Reactive	Non-reactive
Dialysis	0	44
SLE	0	56
Pregnant Women	0	59
Malignant Tumor	0	59
Elderly (Age: 69-91)	0	49
Rheumatoid Factor Positive	0	48
Multipara	0	8
*Borrelia burgdorferi*	0	16
Anti-HIV Positive	0	8
Anti-EBV Positive	0	5
Anti-VZV Positive	0	4
Anti-HSV 1/2 Positive	0	5
Anti-HAV Positive	0	5
Anti-HCV Positive	0	5
Anti-HEV Positive	0	5
Anti-CMV Positive	0	5
Antiphospholipid-Ak	0	8
Hyper IgG	0	7
Hyper IgM	0	9
Total	0	405
Analytical Specificity	100.00%	
95% CI	99.06~100.00%	

HIV, human immunodeficiency virus; EBV, Epstein-Barr virus; VZV, varicella-zoster virus; HSV, herpes simplex virus; HAV, hepatitis A virus; HCV, hepatitis C virus; HEV, hepatitis E virus; CMV, cytomegalovirus; SLE, systemic lupus erythematosus; CI, confidence interval.

### 3.3 Analytical sensitivity at the cutoff

The dilution series of the NIBSC 05/132 standard was tested in parallel on the MAGLUMI Syphilis (CLIA), ARCHITECT TP and Elecsys Syphilis. The obtained results are summarized in **Table 4**. When the cutoff value of the MAGLUMI Syphilis (CLIA) Test is set at 1 AU/mL, the concentration of the NIBSC 05/132 standard is 2.529 mIU/mL.

**Table 4:** Analytical sensitivity of MAGLUMI syphilis (CLIA) test in samples with international standard.

NIBSC 05/132 concentration (mIU/ml)	MAGLUMI Syphilis (CLIA) (AU/ml)	ARCHITECT Syphilis TP (S/CO)	Elecsys Syphilis (COI)
300	152	15.68	29.45
75	32.4	5.43	7.54
18.8	6.83	1.26	1.99
4.7	1.77	0.32	0.61
1.2	0.516	0.07	0.251
Blank	0.207	0.04	0.103

### 3.4 Typical distribution of values

The MAGLUMI syphilis test shows good discrimination between reactive and non-reactive samples. In the MAGLUMI syphilis assay, only a few samples had low positive AU/ML values (n = 6186; 16 samples had AU/ML values ranging from 1 to 5, representing 0.25%). These weakly reactive samples were probably early infections due to the positive results identified by both Abbott ARCHITECT Syphilis TP and Roche Elecsys Syphilis. However, we did not conduct follow-up to confirm syphilis infection, so we cannot completely rule out false positive results and further research is still needed to evaluate the performance of the test in the weakly positive samples. All other samples from patients with syphilitic disease had AU/mL values ranging from 5.53 to ≥600.

## 4 Discussion

This study evaluates the sensitivity and specificity of the MAGLUMI Syphilis (CLIA) test for the MAGLUMI series of fully automated chemiluminescence immunoanalyzers. The MAGLUMI Syphilis (CLIA) test has a 99.96% (5697/5699) specificity for blood donor and inpatient samples and a 100.00% (487/487) sensitivity for syphilis positive samples. In addition, we observed excellent analytical sensitivity by assessing the influence of factors such as interfering substances, complement interference (data not shown), and serum-plasma equivalence. Based on these results, it can be concluded that the MAGLUMI Syphilis (CLIA) test has fairly good specificity and sensitivity.

Primary and secondary syphilis are the two main prevalent syphilis stages globally, and with the use of penicillin in the early stages, the incidence of tertiary syphilis has decreased significantly (Peeling et al., 2017). In this study, 103 samples of known stages of syphilis, including three stage types except latent syphilis. The incidence of primary and secondary syphilis among MSM in the U.S. was 167.5 times that of women and 106 times that of heterosexual men (de Voux et al., 2017). Syphilis is 300 times more common among HIV-positive MSM compared with cases reported in the general male population (Burchell et al., 2015). Treponemal tests are generally more sensitive than nontreponemal tests in detecting early syphilis and can identify the treatment status of syphilis (Castro et al., 2013; Janier et al., 2014). However, it may produce a considerable number of false-positive results in populations with a low prevalence of syphilis (Janier et al., 2014). In this study, Snibe’s syphilis test is a chemiluminescent immunoassay for the detection of *T. pallidum*, which has the advantage of high sensitivity. All 103 samples of syphilis at different stages (49 cases of primary syphilis, 19 cases of secondary syphilis, and 35 cases of tertiary syphilis) were accurately diagnosed as positive, achieved a sensitivity of 100.00%. The positive predictive value in this study also reached 99.59%, but the positive predictive value in low-prevalence areas needs further study.

Pregnant women should be tested for syphilis at the first prenatal care visit and treated right away if the test result is positive. Congenital syphilis can only be prevented by treating the mother with penicillin (Janier et al., 2021). In addition, studies have found a relatively high prevalence of false-positive syphilis biological reactions in patients with autoimmune diseases, pregnancy, infectious diseases, and malignancies (Ishihara et al., 2021). we evaluated clinical performance of the test in 315 particular populations including 59 pregnancy, 49 elder people, 44 dialysis patients, 59 patients with malignancies and 104 people with autoimmunity disease (56 systemic lupus erythematosus and 48 rheumatoid factor positive patients) and no cross reactivity occurred (**Table 3**). In some cases, false-positive treponemal tests can be seen with other conditions including other spirochetal infections, malaria, and leprosy (Golden et al., 2003). On the other hand, for nontreponemal antigen-based tests, conditions such as other infections (e.g., HIV), autoimmune conditions, vaccinations, injecting drug use, pregnancy, and older age can also cause false positives (Nandwani and Evans, 1995; Tuddenham et al., 2020). Lyme disease is another infectious disease caused by the Spirochaetaceae family, which may be potential to impact syphilis testing. However, one study has shown that the false-positive rate of syphilis testing was very low in samples from patients with a history of Lyme disease (Patriquin et al., 2016). It is worth noting that this study also included 16 samples of Lyme disease patients whose sera containing antibodies to *Borrelia burgdorferi* and no cross reactivity was observed (**Supplementary Table 2**). However, whether there might be sera reactive to other Lyme spirochetes needs to be further evaluated.

Several studies reported that using of treponemal screening assay strength of signal may avoid unnecessary confirmatory testing to improve reverse screening algorithm for *T. pallidum* antibody (Yen-Lieberman et al., 2011; Dai et al., 2014; Berry and Loeffelholz, 2016). However, more CLIA reactive but *T. pallidum* particle agglutination test (TPPA) nonreactive specimens are needed to determine reliable AU/mL and index values to use clinically.

At present, there are still some shortcomings in the methods for syphilis detection, such as high cost and complicated operation. Therefore, it is still necessary to optimize the existing tests or develop new ones. The MAGLUMI Syphilis (CLIA) Test is a chemiluminescence immunoassay (CLIA). Performance comparisons between different CLIAs with various assay formats showed great agreement for syphilis detection (Adhikari et al., 2020). Compared with the traditional enzyme-linked immunosorbent assay (ELISA) test, CLIA has shown comparable or even superior performance in detecting serum *T. pallidum* specific antibodies, with higher reliability, sensitivity, accuracy, and rapidity (Li et al., 2016). Rapid point of care tests (POCTs) for syphilis are highly beneficial in areas with scarce resources, as they enhance access to screening and treatment, thereby reducing the risk of stillbirths and neonatal mortality associated with congenital syphilis (Li et al., 2016). A meta-analysis has shown that the sensitivity of commonly used POCTs varies between 74.48% and 90.04% in serum and between 74.26% and 86.32% in whole blood (Jafari et al., 2013). Additionally, one study reported that a POCT based on colloidal gold method using the same antigens perform a sensitivity of 82% (95% CI, 68-91%) (Yang et al., 2010), which is lower than MAGLUMI Syphilis (CLIA) Test. It is worth mentioning that the MAGLUMI platform has also developed the small-sized MAGLUMI X3 (0.68 m^2^), suitable for working environments with constrained resources. In conclusion, the MAGLUMI Syphilis (CLIA) test shows accurate and reliable clinical performance in detecting antibodies to syphilis and can provide strong support for syphilis screening.

## Data Availability

The raw data supporting the conclusions of this article will be made available by the authors, without undue reservation.

## References

[B1] Adhikari E. H. Frame I. J. Hill E. Fatabhoy R. Strickland A. L. Cavuoti D.. (2020). Abbott ARCHITECT syphilis TP chemiluminescent immunoassay accurately diagnoses past or current syphilis in pregnancy. Am. J. Perinatol 37, 112–118. doi: 10.1055/s-0039-3400994 31905408

[B2] Avila-Nieto C. Pedreno-Lopez N. Mitja O. Clotet B. Blanco J. Carrillo J. (2023). Syphilis vaccine: challenges, controversies and opportunities. Front. Immunol. 14. doi: 10.3389/fimmu.2023.1126170 PMC10118025 37090699

[B3] Berry G. J. Loeffelholz M. J. (2016). Use of treponemal screening assay strength of signal to avoid unnecessary confirmatory testing. Sex Transm Dis. 43, 737–740. doi: 10.1097/OLQ.0000000000000524 27835625

[B4] Burchell A. N. Allen V. G. Gardner S. L. Moravan V. Tan D. H. Grewal R.. (2015). High incidence of diagnosis with syphilis co-infection among men who have sex with men in an HIV cohort in Ontario, Canada. BMC Infect. Dis. 15, 356. doi: 10.1186/s12879-015-1098-2 26289937 PMC4546079

[B5] Jost H. Castro A. Cox D. Fakile Y. Kikkert S. Tun Y.. (2013). A comparison of the analytical level of agreement of nine treponemal assays for syphilis and possible implications for screening algorithms. BMJ Open 3, e003347. doi: 10.1136/bmjopen-2013-003347 PMC3780299 24056483

[B6] Dai S. Chi P. Lin Y. Zheng X. Liu W. Zhang J.. (2014). Improved reverse screening algorithm for Treponema pallidum antibody using signal-to-cutoff ratios from chemiluminescence microparticle immunoassay. Sex Transm Dis. 41, 29–34. doi: 10.1097/OLQ.0000000000000066 24326578

[B7] de Voux A. Kidd S. Grey J. A. Rosenberg E. S. Gift T. L. Weinstock H.. (2017). State-specific rates of primary and secondary syphilis among men who have sex with men - United States, 2015. MMWR Morb Mortal Wkly Rep. 66, 349–354. doi: 10.15585/mmwr.mm6613a1 28384130 PMC5657910

[B8] Golden M. R. Marra C. M. Holmes K. K. (2003). Update on syphilis: resurgence of an old problem. JAMA 290, 1510–1514. doi: 10.1001/jama.290.11.1510 13129993

[B9] Ishihara Y. Okamoto K. Shimosaka H. Ono Y. Kanno Y. Ikeda M.. (2021). Prevalence and clinical characteristics of patients with biologically false-positive reactions with serological syphilis testing in contemporary practice: 10-year experience at a tertiary academic hospital. Sex Transm Infect. 97, 397–401. doi: 10.1136/sextrans-2020-054628 33208510

[B10] Jafari Y. Peeling R. W. Shivkumar S. Claessens C. Joseph L. Pai N. P. (2013). Are Treponema pallidum specific rapid and point-of-care tests for syphilis accurate enough for screening in resource limited settings? Evidence from a meta-analysis. PloS One 8, e54695. doi: 10.1371/journal.pone.0054695 23468842 PMC3582640

[B11] Janier M. Hegyi V. Dupin N. Unemo M. Tiplica G. S. French P.. (2014). 2014 European guideline on the management of syphilis. J. Eur. Acad. Dermatol. Venereol 28, 1581–1593. doi: 10.1111/jdv.12734 25348878

[B12] Janier M. Unemo M. Dupin N. Tiplica G. S. Potocnik M. Patel R. (2021). 2020 European guideline on the management of syphilis. J. Eur. Acad. Dermatol. Venereol 35, 574–588. doi: 10.1111/jdv.16946 33094521

[B13] Jankowska L. Adamski Z. Polanska A. Bowszyc-Dmochowska M. Plagens-Rotman K. Merks P.. (2022). Challenges in the diagnosis of tertiary syphilis: case report with literature review. Int. J. Environ. Res. Public Health 19, 16992. doi: 10.3390/ijerph192416992 36554872 PMC9778711

[B14] Kenyon C. Lynen L. Florence E. Caluwaerts S. Vandenbruaene M. Apers L.. (2014). Syphilis reinfections pose problems for syphilis diagnosis in Antwerp, Belgium - 1992 to 2012. Euro Surveill 19, 20958. doi: 10.2807/1560-7917.ES2014.19.45.20958 25411690

[B15] Kingston M. French P. Higgins S. McQuillan O. Sukthankar A. Stott C.. (2016). UK national guidelines on the management of syphilis 2015. Int. J. STD AIDS 27, 421–446. doi: 10.1177/0956462415624059 26721608

[B16] Li L. Cai B. Tao C. Wang L. (2016). Performance evaluation of CLIA for treponema pallidum specific antibodies detection in comparison with ELISA. J. Clin. Lab. Anal. 30, 216–222. doi: 10.1002/jcla.21839 25716172 PMC6807082

[B17] Nandwani R. Evans D. T. (1995). Are you sure it’s syphilis? A review of false positive serology. Int. J. STD AIDS 6, 241–248. doi: 10.1177/095646249500600404 7548285

[B18] Papp J. R. Park I. U. Fakile Y. Pereira L. Pillay A. Bolan G. A. (2024). CDC laboratory recommendations for syphilis testing, United States, 2024. MMWR Recomm Rep. 73, 1–32. doi: 10.15585/mmwr.rr7301a1 PMC10849099 38319847

[B19] Park I. U. Tran A. Pereira L. Fakile Y. (2020). Sensitivity and specificity of treponemal-specific tests for the diagnosis of syphilis. Clin. Infect. Dis. 71, S13–S20. doi: 10.1093/cid/ciaa349 32578866 PMC7312216

[B20] Patriquin G. LeBlanc J. Heinstein C. Roberts C. Lindsay R. Hatchette T. F. (2016). Cross-reactivity between Lyme and syphilis screening assays: Lyme disease does not cause false-positive syphilis screens. Diagn. Microbiol Infect. Dis. 84, 184–186. doi: 10.1016/j.diagmicrobio.2015.11.019 26707064

[B21] Peeling R. W. Mabey D. Chen X. S. Garcia P. J. (2023). Syphilis. Lancet 402, 336–346. doi: 10.1016/S0140-6736(22)02348-0 37481272

[B22] Peeling R. W. Mabey D. Kamb M. L. Chen X. S. Radolf J. D. Benzaken A. S. (2017). Syphilis. Nat. Rev. Dis. Primers 3, 17073. doi: 10.1038/nrdp.2017.73 29022569 PMC5809176

[B23] Qin J. Yang T. Xiao S. Tan H. Feng T. Fu H. (2014). Reported estimates of adverse pregnancy outcomes among women with and without syphilis: a systematic review and meta-analysis. PloS One 9, e102203. doi: 10.1371/journal.pone.0102203 25025232 PMC4099012

[B24] Rac M. W. Revell P. A. Eppes C. S. (2017). Syphilis during pregnancy: a preventable threat to maternal-fetal health. Am. J. Obstet Gynecol 216, 352–363. doi: 10.1016/j.ajog.2016.11.1052 27956203

[B25] Sankaran D. Partridge E. Lakshminrusimha S. (2023). Congenital syphilis-an illustrative review. Children (Basel) 10 (8), 1310. doi: 10.3390/children10081310 37628309 PMC10453258

[B26] Silva A. A. O. de Oliveria U. D. Vasconselos L. C. M. Foti L. Leony L. M. Daltro R. T.. (2020). Performance of Treponema pallidum recombinant proteins in the serological diagnosis of syphilis. PloS One 15, e0234043. doi: 10.1371/journal.pone.0234043 32555593 PMC7302711

[B27] Trinh T. Leal A. F. Mello M. B. Taylor M. M. Barrow R. Wi T. E.. (2019). Syphilis management in pregnancy: a review of guideline recommendations from countries around the world. Sex Reprod. Health Matters 27, 69–82. doi: 10.1080/26410397.2019.1691897 31884900 PMC7888020

[B28] Tuddenham S. Katz S. S. Ghanem K. G. (2020). Syphilis laboratory guidelines: performance characteristics of nontreponemal antibody tests. Clin. Infect. Dis. 71, S21–S42. doi: 10.1093/cid/ciaa306 32578862 PMC7312285

[B29] WHO (2021).Global progress report on HIV, viral hepatitis and sexually transmitted infections. Available online at: https://www.who.int/publications/i/item/9789240027077 (Accessed 24 June 2023).

[B30] WHO. (2022). Global health sector strategies on, respectively, HIV, viral hepatitis and sexually transmitted infections for the period 2022-2030. Chapter 6, 76-77. Available online at: https://www.who.int/publications/m/item/global-health-sector-strategies-on-respectively--hiv-viral-hepatitis-and-stis-for-2022-2030 (Accessed June 5, 2022).

[B31] WHO (2023). Syphilis. Available online at: https://www.who.int/news-room/fact-sheets/detail/syphilis (Accessed May 21, 2024).

[B32] Yang H. Li D. He R. Guo Q. Wang K. Zhang X.. (2010). A novel quantum dots-based point of care test for syphilis. Nanoscale Res. Lett. 5, 875–881. doi: 10.1007/s11671-010-9578-1 20672123 PMC2893857

[B33] Yen-Lieberman B. Daniel J. Means C. Waletzky J. Daly T. M. (2011). Identification of false-positive syphilis antibody results using a semiquantitative algorithm. Clin. Vaccine Immunol. 18, 1038–1040. doi: 10.1128/CVI.05066-11 21508162 PMC3122602

