# Supplementary material for: Evaluation of MAGLUMI syphilis test for accurate detection of syphilis antibodies in blood donors and suspected syphilis cases
Source: Front Cell Infect Microbiol. 2025 May 15;15:1578060. doi: 10.3389/fcimb.2025.1578060 (PMC12119618; doi:10.3389/fcimb.2025.1578060)
Supplement: Supplementary file 1 [file Table1.docx]

Supplementary Table 1. Summary of results for two discordant donor samples.

| Donor Sample ID |  | MAGLUMI Syphilis (CLIA) (Cutoff = 1 AU/mL) | | | | Elecsys Syphilis (Cutoff = 1 COI) | | | | | | ARCHITECT Syphilis TP (Cutoff = 1 S/CO) | | | | | | |
| --- | --- | --- | --- | --- | --- | --- | --- | --- | --- | --- | --- | --- | --- | --- | --- | --- | --- | --- |
|  |  | | Results | Interpretation | | | Results | Interpretation | | | Results | | | | Interpretat  ion | | | |
| 1200792690 | Initial | 1.06 | | Positive | | | 0.679 | Negative | | | 0.31 | | | | Negative | | | |
|  | Repeat 1 | 1.09 | |  | | |  |  | | |  | | | |  | | | |
|  | Repeat 2 | 1.09 | |  |  |  |  |  |  |  |  |  |  |  |  |  |  |  |
| 1200918240 | Initial | 1.08 | | Positive | | | 0.281 | Negative | | | 0.20 | | | | Negative | | | |
|  | Repeat 1 | 1.13 | |  | | |  | | |  | | | |  | | |  |  |
|  | Repeat 2 | 1.17 | |  |  | | | |  | | | |  | | |  | | |

Supplementary Table 2. Detection results in Anti-*Borrelia burgdorferi* positive samples.

|  |  |  | |  | MAGLUMI Syphilis (CLIA) (Cutoff = 1 AU/mL) | | ARCHITECT Syphilis TP (Cutoff = 1 S/CO) | | |
| --- | --- | --- | --- | --- | --- | --- | --- | --- | --- |
| Sample ID | Gender | Age | collection date | | Results | Interpretation | Results | Interpretation |  |
| 63830490  63865553  66055995  63966488  63970763  64010618  66060190  64064997  63923986  64048165  63871451  63838226  66031150  63924173  64160326  64150734 | Male  Male  Male  Female  Male  Male  Female  Male  Male  Female  Male  Female  Male  Male  Male  Male | 23  26  28  21  23  62  24  23  21  45  33  22  33  24  35  43 | 27/04/2018  16/07/2018  24/10/2019  01/04/2019  11/04/2019  08/01/2014  05/11/2019  24/09/2019  07/12/2018  26/07/2018  26/07/2018  16/05/2018  23/08/2019  07/12/2018  11/03/2022  16/11/2021 | | 0.137  0.154  0.103  0.141  0.146  0.182  0.094  0.144  0.134  0.170  0.145  0.143  0.140  0.161  0.135  0.122 | Negative  Negative  Negative  Negative  Negative  Negative  Negative  Negative  Negative  Negative  Negative  Negative  Negative  Negative  Negative  Negative | 0.07  0.04  0.03  0.05  0.03  0.05  0.06  0.03  0.11  0.11  0.02  0.05  0.02  0.04  0.08  0.03 | Negative  Negative  Negative  Negative  Negative  Negative  Negative  Negative  Negative  Negative  Negative  Negative  Negative  Negative  Negative  Negative |  |
